# Epidemiology of proximal humerus fractures in Qatar

**DOI:** 10.1007/s00590-023-03539-5

**Published:** 2023-04-10

**Authors:** Osama Z. Alzobi, Loay A. Salman, Jawad Derbas, Abedallah Abudalou, Ashraf T. Hantouly, Ghalib Ahmed

**Affiliations:** Department of Orthopaedic Surgery, Surgical Speciality Center, Hamad Medical Corporation, Doha, Qatar

**Keywords:** Proximal, Humerus, Fracture, Qatar, Epidemiology, Incidence

## Abstract

**Objective:**

This study aimed to investigate the epidemiological characteristics and treatment options of proximal humerus fractures at a level one trauma center and to compare our data with the current literature.

**Methods:**

A retrospective review was conducted on all patients diagnosed and treated for proximal humerus fractures at Hamad General Hospital, a level one trauma center, between January 2018 and December 2019. Age, gender, mechanism of injury, fracture classification, mode of treatment, implant type, length of hospital stay, associated injuries and complications were analyzed.

**Results:**

A total of 190 patients with a mean age of 52.4 years were included; 56.8% were males. The incidence of proximal humerus fracture was 4.1/100,000 per year. Falling from a standing height was the most common cause of injury (50.5%). Additionally, Neer’s two-part fracture was found to be the most common type (*n* = 132, 69%). Forty-one patients (21.3%) had other associated injuries. Most fractures were treated nonoperatively with an arm sling (*n* = 138, 72.6%).

**Conclusion:**

In summary, the incidence of proximal humerus fractures during the two-year study period was found to be 4.1 per 100,000 persons per year. Our results showed a lower incidence of proximal humerus fractures with a predominance of males and younger patients in Qatar’s population compared to females and older patients in the developed countries. Our results may contribute to the development of effective strategies for preventing and treating proximal humerus fractures, and can provide important data for further high-level clinical research.

**Level of evidence:**

IV.

## Introduction

Proximal humerus fractures (PHFs) are common injuries and account for 5–6% of all adult fractures [1]. These fractures have a bimodal distribution that is influenced by age and energy level; young adults sustain fractures due to high-energy trauma, while the elderly population sustains these fractures due to low-energy trauma [2]. These fractures are the third most common fragility fractures after distal radius and hip fractures, which implies that aging and osteoporotic bone have a primary role in PHFs in the elderly [2, 3]. Additionally, the increase could also be expected in elderly individuals due to improved healthcare systems, as shown by the steady rise in life expectancy of the population of Qatar from 55 to 80 years since the 1950s [4]. The longevity of life expectancy is comparable to the most developed countries that have improved life expectancy, with the highest average of 85 years [4]. It is worth mentioning that the most common mechanisms of traumatic injuries in the Arab Middle Eastern region include road traffic accidents, falls from height, falls of heavy objects, and pedestrian injuries [5, 6].

Qatar has grown substantially in the past few years, economically and technologically. The thriving construction industry in Qatar has led to a further increase in the growth of the population, number of motor vehicles, and workers, which may increase the incidence and prevalence of PHFs in young adults.

PHFs may impact the patient’s life medically, socially, and financially. The high costs associated with acute hospitalization and long-term rehabilitation of PHFs may also imply a financial burden on the healthcare system [6].

This study aimed to investigate the epidemiology of PHFs, including incidence rates, patient characteristics, fracture morphology, and treatment methods, and compare these data with the available records in the literature.

## Materials and methods

A retrospective analysis was undertaken on all patients diagnosed and treated for PHFs treated in Qatar between January 2018 and December 2019. The institutional review board at our institution approved the study.

Data were retrieved from medical records and radiology archives were assessed to identify patients diagnosed with PHFs. All patients above the age of 18 years with PHFs were included and radiographs were assessed to exclude any miscoded diagnoses for all cases. Data for essential details, including age, gender, the cause of injury, associated shoulder dislocations, nerve injuries, other associated injuries, the nature of the treatment, and complications were recorded.

Data about treatment and post-treatment complications were recorded in a computer database for subsequent analysis. Fractures were classified according to Neer’s classification by senior surgeons using anterior–posterior and scapula Y radiographs.

For statistical analysis, categorical and continuous data were expressed in percentage, mean, and range and were correlated using percentages and graphical comparisons.

Descriptive statistics were used to summarize the demographic, classification, treatment methods, and complications. All statistical analyses were performed using the statistical packages SPSS 22.0 (SPSS Inc. Chicago, IL) and Epi Info TM 2000 (Centers for Disease Control and Prevention, Atlanta, GA).

## Results

### Incidence

A total of 190 patients with PHFs admitted to our institution were included. Based on the fact that Hamad Medical Corporation is the only medical institute providing medical care for such fractures for a local population of 2,314,557, the estimated annual incidence of PHFs was 3.8/100,000 per year in 2018 (*n* = 87 cases) and 4.45/100,000 per year in 2019 (*n* = 103 cases).

### Trends in age and gender

The mean age of patients sustaining a PHF between 2018 and 2019 was 52.4 years (range = 20–103 years), with a slight male predominance (*n* = 108, 56.8%). Patients were distributed according to age group into seven age groups, as shown in Table 1.

**Table 1:** Data demographic and clinical information

Total number	Gender		Average age years (Range)	Treatment	
	Male	Female		Non-operative	Operative
190	108	82	52.4 (20–103)	138	52

**Fig. 1:**
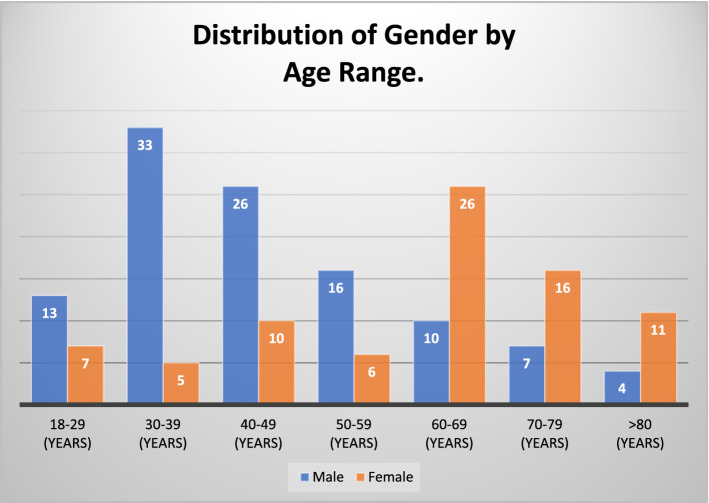
Distribution of gender by age range

Analysis by age group showed a higher incidence of fractures in men between 30 and 39 and 40–49 years, along with women aged between 60 and 69 years, as shown in Fig. 1.

### Mechanism of injury

**Fig. 2:**
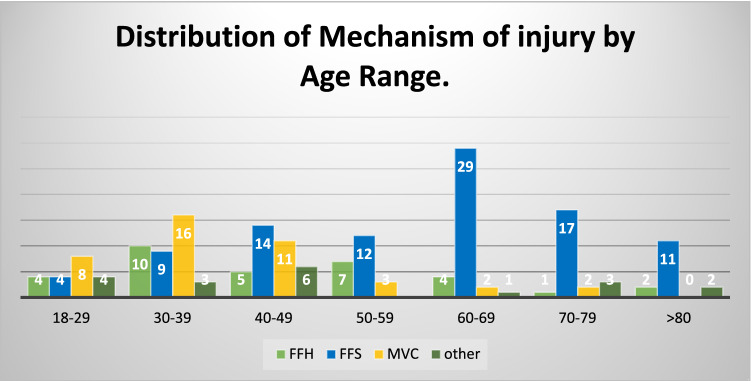
Distribution of mechanism of injury by age range. (FFH: Fall from height, FFS: Fall from standing height, MVC: Motor vehicle collision)

The most common cause of injury in all age groups was falling from standing height (FFS) (50.5%), followed by motor vehicle collisions (MVC) (18.4%), falling from height (FFH) (17.4%), pedestrians struck by vehicles (2.8%), and seizures without other contributory causes (2.8%). The most common cause of injury in elderly groups (age over 60 years) was FFS (77%; *n* = 57 patients). MVCs were more common in the younger age group (30–39 and 40–49 years) than in other age groups, as shown in Table 2 and Fig. 2.

**Table 2:** A summary of different mechanisms of injury for all proximal humerus fractures: (FFH: Fall from height, FFS: Fall from standing height, MVC: Motor vehicle collision). Others include three patients due to seizures, four due to sport-related activities and three due to direct traumatic injuries at work

Mechanism of injury	No of Patients	%
FFH	33	17.4
FFS	96	50.5
MVC	42	22.1
Other	19	10
Total	190	100

### Types of fracture

**Fig. 3:**
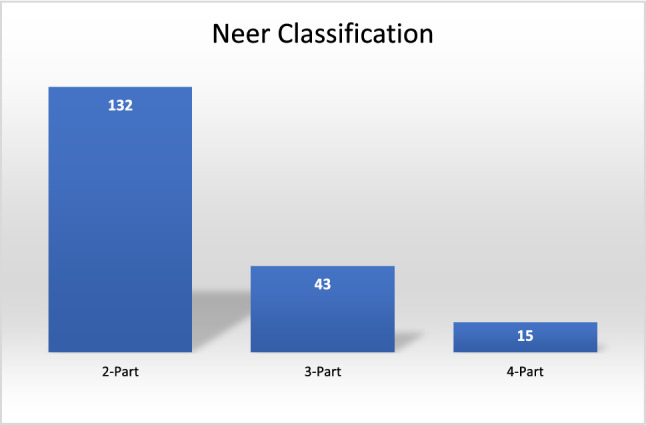
Fractures classification according to Neer’s classification system

The Neer classification system was used to describe the number of fracture parts in PHFs. Of all PHFs, 69% were two-part fractures and 22.6% were three-part fractures, as shown in Fig. 3. The AO classification system was used to describe the morphology of PHFs. Type A fractures were the most common, accounting for 60% of all fractures. A1 fractures, which involve the greater tuberosity fractures, accounted for 25.3% of all PHFs. A2 and A3 fractures (involving the surgical neck), accounted for 26.3% and 8.4% of all PHFs, respectively. Type B fractures, which are bifocal fractures or fracture-dislocations, accounted for 28.8% of all PHFs. B1 fractures (impacted bifocal fractures) accounted for 14.7% of all PHFs, while B2 fractures (non-impacted bifocal fractures) and B3 (bifocal fracture-dislocations) accounted for 8.4% and 5.7% of all PHFs, respectively. Finally, 11.2% of all PHFs were classified as type C fractures, which are intra-articular anatomical neck fractures.

### Other associated injuries and comorbidities

Over the two years, 21.6% of the cases (*n* = 41) had polytrauma-associated injuries, which included long bone fractures, chest, head, and abdominal injuries. The majority of these injuries (80%) occurred in patients under the age of 60. Additionally, 81 cases had ipsilateral limb injuries, and five were open fractures. Most of these injuries (70%) also occurred in patients under the age of 60.

Among the diseases reported by the patients, systemic hypertension and diabetes were the most prevalent, with 59 cases (31%) for each disease. The prevalence of coronary artery disease was 7.9%, high cholesterol was 6.8%, osteoporosis was 4.7%, and seizure disorders were 4.7%.

### Treatment options

Non-operative treatment was considered for 72.6% (*n* = 138 cases) of all PHFs, with a lower rate of operations in the elderly. Non-operative treatment involved immobilization with a simple sling.

Operative treatment was considered for 27.4% (*n* = 52 cases) of PHFs. Open reduction with a locking plate was used for 73% (*n* = 38 cases) of patients who underwent surgery. Open reduction with sutures or screws fixation was used for five cases. Intramedullary nailing was used for two cases and K-wires fixation was used for two cases. Three patients underwent hemiarthroplasty and two underwent reverse shoulder arthroplasty. A summary of different treatment options is shown in Table 3.

**Table 3:** A summary of different treatment options

Treatment options			
Non-operative, percentage (*n*)	72.6% (138)	Operative, percentage (*n*)	27.4% (52)
Arm sling	138	Open reduction with a locking plate	38
		Open reduction with sutures or screws	5
		Intramedullary nailing	2
		K-wires fixation	2
		Hemiarthroplasty	3
		Reverse shoulder arthroplasty	2
Total (*n*)	138	Total (*n*)	52

### Complications

The non-operative treatment of 138 patients resulted in some complications. Four patients experienced nonunion (2.9%), three experienced malunion (2.2%), and one experienced avascular necrosis. Additionally, two patients with symptomatic nonunion required conversion to surgical treatment with open reduction internal fixation with bone grafting.

Among the patients who underwent surgery, nonunion occurred in four patients, axillary nerve injury in three patients and resolved in one patient after six months. Implant failure occurred in three patients, deep infection in two and superficial infection in two other patients. 6 patients (11.5%) required a second operation; two of these patients underwent revision surgery due to deep infection, two were due to implant failure and one was due to symptomatic nonunion. One patient required manipulation under anesthesia to address post-operative stiffness. A summary of complications with non-operative and operative treatments is shown in Table 4.

**Table 4:** A summary of complications with non-operative and operative treatments

Complications			
Non-operative treatment	Percentage (*n*)	Operative treatment	Percentage (*n*)
Nonunion	2.9% (4)	Nonunion	7.7% (4)
Malunion	2.2% (3)	Axillary nerve injury	5.8% (3)
Avascular necrosis	0.7% (1)	Implant failure	5.8% (3)
Conversion to surgical treatment	1.4% (2)	Deep Infection	3.8% (2)
Total		Superficial Infection	3.8% (2)
		Second operation	11.5% (6)
	7.2% (10)	Total	38.4% (20)

Two patients with symptomatic nonunion required conversion to surgical treatment. Six patients required a second operation; two due to deep infection, two due to implant failure, one due to symptomatic nonunion and one due to post-operative stiffness

## Discussion

This study highlighted and assessed the epidemiology of PHFs in the young, active, and expanding population of Qatar over a two-year period. The essential ascertainment of our study found that the overall incidence of PHFs was 3.8 per 100,000 cases in 2018 and 4.45 per 100,000 cases in 2019. These figures are lower than the incidence rates reported in previous studies, which ranged from 9.49 to 15.45 per 100,000 cases [7–10].

While similar studies have been conducted before, to our best knowledge, this study remains the first to report a detailed profile of PHFs in the Middle East region. Unlike the previous literature [3–5], this study found that males were more commonly affected than females, with a ratio of 56.8%–43.2%. The mean age of patients included in this study was 52.4 years, which is much younger than the average age reported in other populations. For example, Court-Brown et al. [1] reported an average age of 64.8 years in the British population, while Olivera et al. [9] reported an average age of 65.2 years in the Brazilian population, and Palvanen et al. [11] reported an average age of 75.5 years in the Finnish population. The fact that 85% of Qatar's population falls within the 15–65 age range [12] supports the idea that the bimodal distribution of PHFs in the population may be due to the different types of trauma experienced by different age and gender groups. Younger males may be more likely to experience high-energy trauma due to their occupation, while older females may be more susceptible to low-energy trauma due to age-related changes in bone density and strength.

The findings of the study suggest that falling from a standing height (FFS) was the most common mode of injury leading to PHFs, especially in older age groups. However, MVCs and FFS were more common in the younger age groups. The finding that some of these injuries were work-related is also notable in our data. These findings are consistent with previous studies conducted by Schwartz et al. [13] and Kim et al. [11], which also reported falling to the ground and road traffic accidents as common modes of injury.

Neer [14] and AO classifications [15] were assigned and utilized to describe fracture complexity. The most frequent fracture patterns observed in the study were Neer two-parts which accounted for 69.4% of cases, and AO type A which accounted for 60% of cases.

The management of PHFs varies widely according to the fracture pattern, characteristics, and patient age. In the current study, more than two third (73%) of PHFs were treated nonoperatively with sling immobilization. This was in line with the current evidence from the literature reporting non-surgical treatment in up to 85% of PHFs, particularly in minimally displaced stable fractures [16, 17]. The surgical treatment options for patients with displaced PHFs reported in this study involved open reduction and locking plate fixation in most of the cases, and 9.6% of operatively treated patients underwent hemiarthroplasty. The landmark PROPHER [18] trial, which was published in 2015, compared surgical and non-surgical treatment options for displaced PHFs involving the surgical neck and found no significant difference in patient-reported outcomes between the two groups. Moreover, the multicentric PROFPHER-2 trial is taking place to evaluate the effectiveness and cost-effectiveness of three treatment options for acute three and four-part fractures of the proximal humerus in patients aged over 65 years [19]. The three treatment options being compared are reverse shoulder arthroplasty, hemiarthroplasty, and non-surgical care.

The complication rates in this study are comparable to those found in the existing literature [20–22]. The incidence of nonunion ranges from 1.1% to 10%, depending on the degree of comminution [21]. In this study, nonunion occurred in 2.9% of the non-operative group and 7.7% in the surgical group. The literature reports a wide range of infection rates for PHFs, from 0 to 10%. Martinez et al. [22] reported two cases of infection out of 18 surgically treated PHF patients, which required revision surgery. In this study, four cases of infection were encountered in the surgical group and two of those cases required revision surgery with implant removal and cement spacers insertion before the final operation after infection eradication.

Owing to the retrospective nature of this study, several limitations must be acknowledged, including the inevitable selection bias. Another limitation was the inclusion of the adult population only. However, proximal humerus epiphysial fractures are not as common and represent only 0.5% of all pediatric fractures [14]. Furthermore, a longer follow-up would have enabled us to better observe the long-term outcomes and potential complications.

## Conclusion

In summary, the incidence of proximal humerus fractures during this two-year study period was 4.1 per 100,000 persons per year. Our results showed a lower incidence of proximal humerus fractures with a predominance of males and younger patients in Qatar’s population compared to females and older patients in the developed countries. Our results may contribute to the development of effective strategies for managing such injuries and provide data for further high-level clinical research.
